# Endoscopic Tissue Adhesive for Gastric Varices

**DOI:** 10.1155/1991/93804

**Published:** 1991

**Authors:** Kaj Johansen

**Affiliations:** Harborview Medical Center Seattle Washington 98104 USA


6. Miller, B.M., Kozarek, R.A., Ryan, J.A., Ball, T.J. and William Traverso, L. (1987) Surgical Versus Endoscopic Management of Common Bile Duct Stones. *Ann. Surg.*, **207**, 135-141
7. Leese, T., Neoptolemos, J.P., Baker, A.R. and Carr-Locke, D.L. (1986) Management of acute cholangitis and the impact of endoscopic sphincterotomy. *Br. J. Surg.*, **73**, 988–992
8. Neoptolemos, J.P. and Carr-Locke, D.L., London, N.J., Bailey, I.A., James, D. and Fossard D.P. (1988) Controlled trial or urgent endoscopic retrograde cholangio-pancreatography and endoscopic sphincterotomy versus conservative treatment for acute pancreatitis due to gallstones. *Lancet* **II**, 979–983
9. Sheridan, W.G., Williams, H.O.L. and Lewis, M.H. (1987) Morbidity and mortality of common bile duct exploration. *Br. J. Surg.*, **74**, 1095–1099
10. Dresemann, G., Kautz, G. and Bünte, H. (1988) Langzeitergebnisse nach endoskopischer Sphinkterotomie bei Patienten mit Gallenblase *in situ. Dtsch. Med. Wschr.*, **113**, 500–505
11. Classen, M., Hagenmüller, F., Knyrim, K. an Frimberger, E. (1988) Giant Bile Duct Stones - Non-Surgical Treatment. *Endoscopy*, **20**, 21-26
12. Cairns, S.R., Dias, L., Cotton, P.B., Salmon, P.R. and Russell, R.C.G. (1989) Additional endoscopic procedures instead of urgent surgery for retained common bile duct stones. *Gut*, **30**, 535–540
13. Dowsett, J.F., Vaira, D., Polydorou, A., Russell, R.O.G. and Salmon, P.R. (1988) Interventional Endoscopy in the Pancreatobiliary Tree. *Am. J. Gastroenterol*, **83**, 1328–1336
14. Davidson, B.R., Neoptolemos, J.P. and Carr-Locke, D.L. (1988) Endoscopic sphincterotomy for common bile duct calculi in patients with gall bladder *in situ* considered unfit for surgery. *Gut*, **29**, 114–120
15. Worthley, C.S. and Toouli, J. (1988) Gallbladder non-filling: an indication for cholecystectomy after endoscopic sphincterotomy. *Br. J. Surg.*, **75**, 796–798

### ENDOSCOPIC TISSUE ADHESIVE FOR GASTRIC VARICES

### ABSTRACT

*Ramond, M-J, Valla, D., Mosnier, J-F, Degott, C., Bernau., J., Rueff, B. and Benhamou, J-P. (1989) Successful Endoscopic Obturation of Gastric Varices with Butyl Cyanoacylate. Hepatology; 10: 488–493.*

**In 27 patients who had bled from esophagogastric varices, large-sized and/or actively bleeding gastric varices were endoscopically obturated with the tissue adhesive butyl cyanoacrylate. Active bleeding was stopped in six patients. Rebleeding occurred in 10 patients; in four patients, rebleeding was due to ruptured gastric varices, occurred early and was successfully treated by reinjection of gastric varices; in one patient, rebleeding was attributed to ulceration on an injected gastric varix. Eight patients died: two of rebleeding (from esophageal varices or undetermined source), four of sepsis and/or liver failure and two at home of undetermined cause. No specific complication due to injection of gastric varices was observed. The results obtained in this series of patients with gastric varices obturated by injection of butyl cyanoacrylate are much more satisfactory than those obtained in previously published series of patients with gastric varices treated by injection of sclerosants.**

**PAPER DISCUSSION**

KEYWORDS: Portal hypertension, sclerotherapy, tissue adhesive, gastric varices

The patient with bleeding gastric varices confronts the clinician with a major management dilemma. Little doubt now exists that, at least acutely, the lugubrious natural history of bleeding *esophageal* varices can be positively affected by injection sclerotherapy, even though technical issues (injection techniques, treatment frequency, choice of sclerosant) remain in dispute. Unfortunately, similar success has not been observed when injection sclerotherapy has been attempted for *gastric* varices. The approach is technically more difficult, early re-bleeding is frequent, and the rapid development of gastric ulcers (which prevent further injections) complicates subsequent management [1,2].

The current study by Ramond and coworkers from the renowned Service d'Hepatologie at Hôpital Beaujon in Clichy is therefore timely and provocative, and worthy of careful consideration. These workers describe the use of the tissue cement butyl cyanoacrylate to occlude gastric varices via trans-endoscopic injection in 27 patients, six of whom were actively bleeding.

While the injection of tissue adhesives into varices (primarily esophageal) is not new, having been reported by these authors [3] and others [4], the special anatomy and behavior of gastric varices[5] make this idea unusually interesting. The reason that injection sclerotherapy of *gastric* varices is so rarely successful is the substantially higher blood flow through these varices, and their stellate, sinusoidal morphology – which together result in rapid dilution and diffusion of the sclerosant[2]. However, injection of a tissue adhesive, which immediately polymerizes *in situ* and "obturates" the varices, is a novel and potentially very important concept.

Several positive findings in support of this treatment for gastric varices are proposed by Ramond and colleagues, including: 1) their ability to halt active gastric variceal hemorrhage (7 episodes in 6 patients); 2) relatively few (6 out of 27 patients) rebleeding episodes from gastric varices; 3) infrequent complications (5 patients) due to gastric ulceration; and 4) no apparent systemic complications specific to injection of gastric varices. These data suggest that injection of tissue adhesive into gastric varices is both relatively safe and effective, at least in the short-term.

However, I have several concerns about these data. First, 85% of these patients (23 of 27) were in Grade A or B according to the classification of Pugh [6]; a one-year survival rate of only 61% (Kaplan-Maier) is unexpectedly low for such a good-risk group of patients (two of whom, by the way, were later cured of portal hypertension either by liver transplantation or portal decompression). Second, while the constant finding of extrusion of the variceal "cast" of tissue adhesive from the stomach mucosa was felt to have led to further re-bleeding in only one patient, *any* mucosal defect in the presence of persistent portal hyperetension must be looked upon with concern, and a higher incidence of re-bleeding from these mucosal rents would not be unsurprising.

Finally, these endoscopists' expertise notwithstanding, several episodes of polymer occlusion of the biopsy channel of the endoscope and the injection needle occurred. While the reader learns no more about this problem, it would seem to be an unacceptably expensive complication (especially regarding the endoscope) in view of the virtual impossibility of removing polymerized tissue adhesive from such sites [7].

Because, until recently, bleeding gastric varices have not been treatable by endoscopic means, our Unit and others have generally considered them to be an indication for surgical intervention. Ramond *et al.* now demonstrate that, by the novel use of the tissue adhesive butyl cyanoacrylate to occlude gastric varices, a rate of control of bleeding from gastric varices equivalent to that for standard injection techniques in esophageal varices can be achieved. This is a praiseworthy and useful observation. However, they conclude from their experience that such treatment provides "...a good alternative to surgery" - a conclusion with which I am less comfortable. A review of Child A and B patients undergoing elective *or* emergency portacaval shunts for bleeding esophageal or gastric varices in our Unit reveals a survival rate of about 90% at one year. Such shunted patients, unlike those treated by *any* form of sclerotherapy, are virtually (>99%) cured of further variceal hemorrhage. Thus, an hypothesis that obturation of gastric varices by tissue adhesive is as good as operation surely awaits a randomized trial.

The results of Ramond and coworkers cause me to believe that, were I to admit myself to Hôpital Beaujon with bleeding gastric varices, I should be happy to have my hemorrhage halted by the intravariceal injection of butyl cyanoacrylate. However, well before my gastric mucosa started to extrude those tissue adhesive casts (have any of those chunks of plastic become impacted at the ileocecal valve?) I would hope to have been seen by a surgeon as well.

Kaj Johansen,
Harborview Medical Center
Seattle, Washington 98104 (USA)

### REFERENCES

1. Williams, R. and Westaby, D. (1986) Endoscopic sclerotherapy for oesophageal varices. *Dig.Dis. Sci.* **31** (Suppl. 9), 108S–121S
2. Trudeau, W. and Prindiville, T. (1986) Endoscopic injection sclerosis in bleeding gastric varices. *Gastrointest. Endosc.* **32**, 264–268
3. Ramond, M.J., Valla, D, Gotlib, J.P. *et al.* (1986) Obturation endoscopique des varices oeso-gastriques par le Bucrylate. I. Etude clinique de 49 malades. *Gastroenterol. Clin. Biol.* **10**, 575–579
4. Gotlib, J.P., Demma, I, Fonsecca, A. *et al.* (1984) Resultats a 1 an du traitement endoscopique electif des hemorragies par rupture de varices oesophagiennes chez le cirrhotique (Abstract). *Gastroenterol. Clin. Biol.* **8**, 133A
5. Mathur, S.K., Dalvi, A.N. and Someshwar, V.*et al.*(1990) Endoscopic and radiological appraisal of gastric varices. *Britt. J. Surg.* **77**, 432–435
6. Pugh, R.N.H., Murray-Lyon, I.M., Dawson, J.L. *et al.* (1973) Transection of the oesophagus for bleeding oesophageal varices. *Brit. J. Surg.*, **60**, 636–649
7. Andreani, T. Letter to the Editor. (1987) *Gastroenterol. Clin. Biol.* **11**, 261-262

